# Identification of Critical Genes and Proteins for Stent Restenosis Induced by Esophageal Benign Hyperplasia in Esophageal Cancer

**DOI:** 10.3389/fgene.2020.563954

**Published:** 2020-12-17

**Authors:** Li Weng, Shanshan Shen, Shaoqiu Wu, Xiang Yin, Bingyan Liu, Mingyi Shang, Xiaoping Zou, Aiwu Mao

**Affiliations:** ^1^Department of Intervention, Tongren Hospital, Shanghai Jiao Tong University School of Medicine, Shanghai, China; ^2^Department of Digestive Medicine, Nanjing Drum Tower Hospital, Nanjing University Medical School, Nanjing, China

**Keywords:** esophageal cancer, esophageal stents placement, esophagus restenosis, sequencing, proteomics

## Abstract

This study was conducted to explore the potential genes and proteins associated with esophagus benign hyperplasia induced by esophageal stents. Five patients with esophageal cancer subjected to esophageal stent placement were enrolled in this study. Long non-coding RNA (lncRNA) sequencing and tandem mass tag quantitative proteomics analysis were performed by using the collected hyperplastic samples and adjacent non-hyperplastic tissues. Differentially expressed (DE) RNAs and proteins were analyzed, followed by functional enrichment analysis, protein-protein interaction (PPI) network analysis, and competitive endogenous RNA (ceRNA) network construction. Venn analysis was performed to extract the overlaps between DE mRNAs and DE proteins and the expression correlations between DE mRNA and proteins were analyzed. Results showed that total 642 DE RNAs (457 mRNA and 185 lncRNAs) and 256 DE proteins were detected. DE mRNAs (such as *MAOB*, *SDR16C5*, and *FOSL1*) were enriched in oxidation-reduction process-associated functions. PPI network was comprised of 175 nodes and 425 edges. VEGFA was a significant node with the highest degree. LncRNA-mRNA network with three subnetworks (C1, C2, C3) was constructed for lncRNAs with more than 15 gene targets. RP11-58O9.2 was a significant lncRNA with the most target genes and RP11-667F14.1 regulated more than 20 targets. *FOSL1* was a common target of the two lncRNAs. Function analysis showed that DE lncRNAs were involved in the HTLV-I infection (RP11-58O9.2 and RP11-667F14.1) and IL-17 signaling pathways (RP11-5O24.1 and RP11-58O9.2). Total 11 DE mRNAs were overlapped with DE proteins, among which MAOB and SDR16C5 showed positive correlations between mRNA and protein expression. Function analysis showed that *MAOB* was enriched in oxidation-reduction process and its protein was closely related with response to lipopolysaccharide. *VEGFA*, *FOSL1*, *MAOB*, *SDR16C5*, RP11-58O9.2, RP11-667F14.1, and RP11-288A5.2 may be served as genetic targets for preventing stent restenosis in esophageal cancer.

## Introduction

Esophageal cancer is one of the most common human cancers globally and a leading cause of cancer-related deaths, with an estimated 400,000 deaths in 2012 (Ferlay et al., 2013). Esophageal cancer is typically asymptomatic and the disease has already progressed by the time the first symptoms appear. Therefore, more than half of the patients with esophageal cancer have advanced disease at the time of diagnosis (Pennathur et al., 2013). Dysphagia is the most common symptom of obstructive esophageal cancer (Mariette et al., 2007), which may lead to malnutrition and eventually result in a poor treatment response and poor prognosis (Mariette et al., 2012).

For patients with dysphagia, esophageal stent placement is a commonly used palliative treatment, which can quickly relieve the obstruction symptoms of patients, maintain oral intake, and reconstruct the gastrointestinal nutrition channel (van Heel et al., 2010a; Van Heel et al., 2010b). However, esophageal stent placement may result in recurrent dysphagia due to stent migration and tissue hyperplasia. Particularly, benign tissue hyperplasia-induced stent restenosis is the most intractable complication with an incidence of up to 46.1% (Hindy et al., 2012). Previous studies have reported that esophagus restenosis after esophageal stent placement is caused by fibroblast proliferation, which stimulates restenosis by delivering growth factors to monocytes (Marzocchi et al., 1991; Albiero et al., 2000; John et al., 2001). It has been reported that stents loaded with ^125^I seeds inhibit fibroblast proliferation compared to conventional stents (Gan et al., 2015). However, experiments using animal models indicated that ^125^I seeds cannot prevent benign tissue hyperplasia-induced stent restenosis (Guo et al., 2007). Therefore, investigating the mechanism of benign hyperplasia-induced stent restenosis is urgently needed to develop effective treatments.

In this study, we enrolled five patients with esophageal cancer who had undergone esophagus ^125^I stent placement and explored the potential genes and proteins associated with esophagus benign hyperplasia induced by esophageal stents. Transcriptome sequencing and tandem mass tag (TMT) quantitative proteomics analyses were performed for hyperplastic tissues and normal tissues of the esophagus wall. Transcriptome and proteomics data were analyzed by bioinformatics methods and validated by reverse transcription (RT)-PCR and western blot analyses.

## Results

### Quality Control and Reference Genome Alignment

After removing low-quality reads, the clean reads were mapped to the human reference genome. The read mapping rate of samples ranged from 91.60 to 94.10%.

### Differential Expression Analysis

**FIGURE 1:**
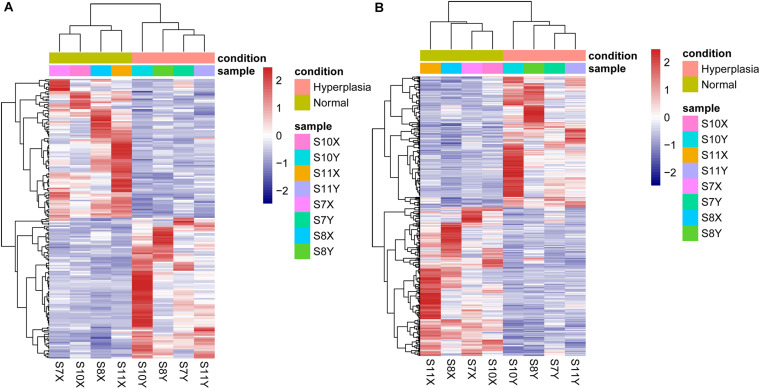
Heatmaps of differentially expressed **(A)** lncRNAs and **(B)** mRNAs.

Using thresholds of |log_2_FC| > 1 and *p* value <0.05, 642 DE genes were identified, including 244 downregulated mRNAs, 213 upregulated mRNAs, 92 downregulated lncRNAs, and 93 upregulated lncRNAs. A heatmap of the DE genes is shown in Figure 1. All DE mRNAs and lncRNAs are shown in Supplementary Table 3.

### Functional Enrichment Analysis of DE mRNAs

**FIGURE 2:**
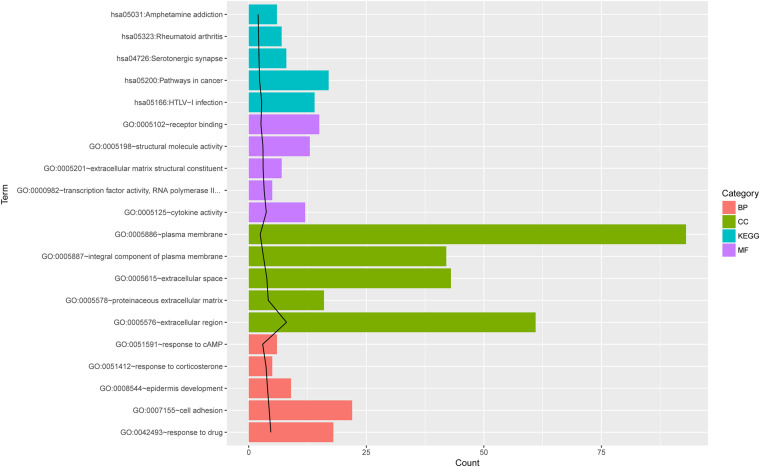
The top 5 GO and KEGG pathway terms for differentially expressed mRNAs. The top five terms ranked according to the increasing *p* values. The black line indicates -log_10_ (*P* value) for each term. BP, biological process; CC, cellar component; MF, molecular function; KEGG, Kyoto Encyclopedia of Genes and Genomes pathway; Count, number of genes enriched in one term.

The DE mRNAs were enriched in 110 GO (BP, CC, and MF) terms or KEGG pathways, such as GO:0007155∼cell adhesion, GO:0005576∼extracellular region, GO:0055114∼oxidation-reduction process, GO:0042542∼response to hydrogen peroxide, and hsa05200:pathways in cancer. The top five terms ranked according to the increasing *p* values are shown in Figure 2.

### PPI Network Construction

In the PPI network of mRNAs, there were 175 nodes and 435 edges. Network topological property analysis revealed that eight mRNAs [such as *VEGFA*, *FOS*, and MYC proto-oncogene, BHLH transcription factor (*MYC*)] had higher scores in three topological properties. The top 15 node genes with higher scores in the three topological properties are shown in Table 1.

**TABLE 1:** Genes with higher degrees in the PPI network (top 15).

Gene	Degree	Gene	Betweenness	Gene	Closeness
VEGFA	39	VEGFA	5672.6035	VEGFA	0.0434674
FOS	36	FOS	4716.4443	FOS	0.0433807
MYC	35	PTGS2	4104.0386	JUN	0.04335908
JUN	35	MYC	3412.1555	MYC	0.04330513
PTGS2	30	JUN	2487.5193	PTGS2	0.04329435
NGF	26	NGF	2372.2073	NGF	0.04313337
EGR1	18	BMP2	2067.0754	SERPINE1	0.04284659
ATF3	17	MUC1	1897.516	MMP7	0.0428255
SERPINE1	15	MMP7	1769.2167	DNAH8	0.04281496
DUSP1	15	GRIN2A	1556.0956	EGR1	0.04278338
CDKN1A	14	DNAH8	1407.1766	BMP2	0.04277286
DNAH8	13	GABRB2	1198	ATF3	0.04273085
AGTR1	13	ALOX12B	1192	DUSP1	0.04272036
BMP2	12	SLC2A1	963.84937	PI3	0.04270987
NR4A2	12	AGTR1	945.83295	HBEGF	0.04270987

### Prediction of Target Genes of DE lncRNAs

**FIGURE 3:**
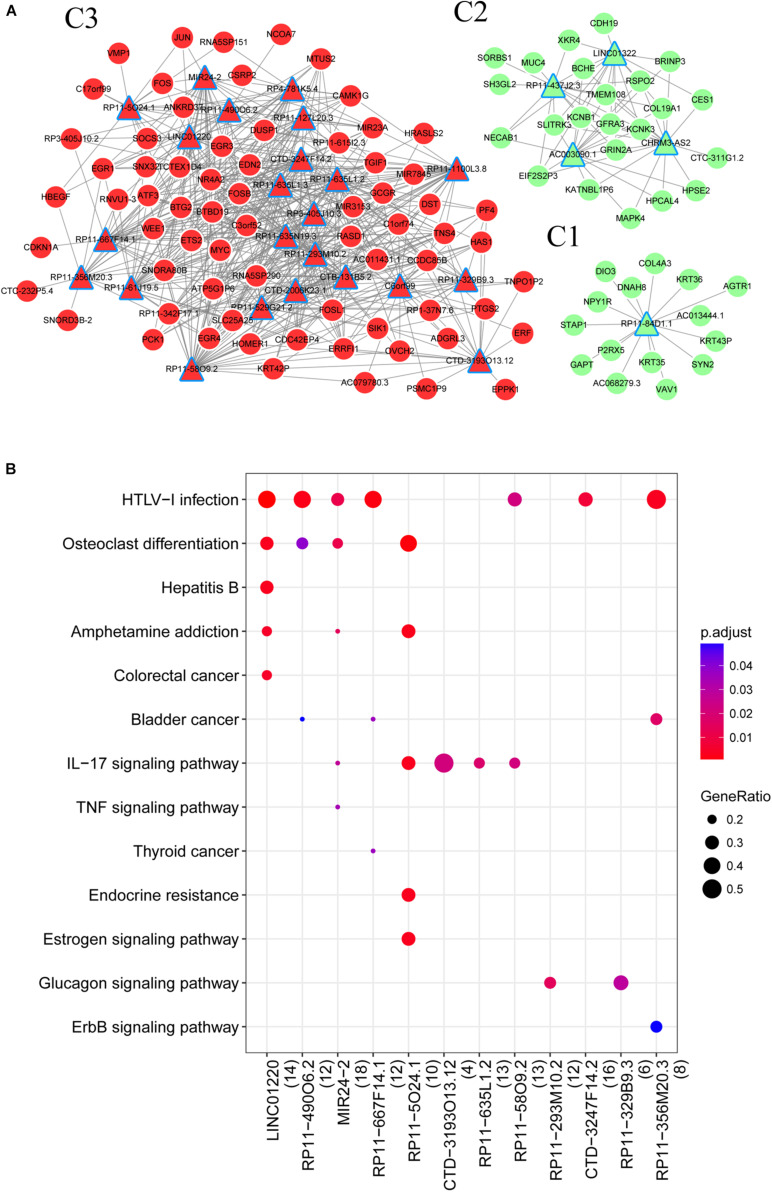
**(A)** LncRNA regulatory network; **(B)** Pathways enriched by lncRNAs. Red triangle, upregulated lncRNA; red circle, upregulated mRNAs; green triangle, downregulated lncRNAs; green circle, downregulated mRNAs.

A total of 151 DE lncRNAs were found to have target genes and lncRNAs that interacted with more than 15 genes were visualized in the lncRNA-mRNA network. As shown in Figure 3A, the network consisted of three parts (C1, C2, and C3), in which there were 1, 4, and 23 lncRNAs, respectively. The top ten lncRNAs that regulated the largest number of targets are shown in Table 2.

**TABLE 2:** The top ten lncRNAs that regulated more targets.

lncRNA	Freq
RP11-58O9.2	36
CTD-3247F14.2	33
RP11-635L1.2	33
RP11-1100L3.8	32
RP11-635N19.3	31
RP11-61J19.5	27
RP11-667F14.1	27
CTB-131B5.2	26
LINC01220	26
MIR24-2	26

To understand the roles of DE lncRNAs, functional enrichment analyses were performed for their target genes. As shown in Figure 3B, the target genes were significantly enriched in the HTLV-I infection (RP11-58O9.2 and RP11-667F14.1) and IL-17 signaling pathways (RP11-5O24.1 and RP11-58O9.2). Additionally, both RP11-58O9.2 and RP11-667F14.1 were associated with functions of response to glucocorticoid, and extracellular stimulus.

### Function Similarity Analysis of lncRNAs

**FIGURE 4:**
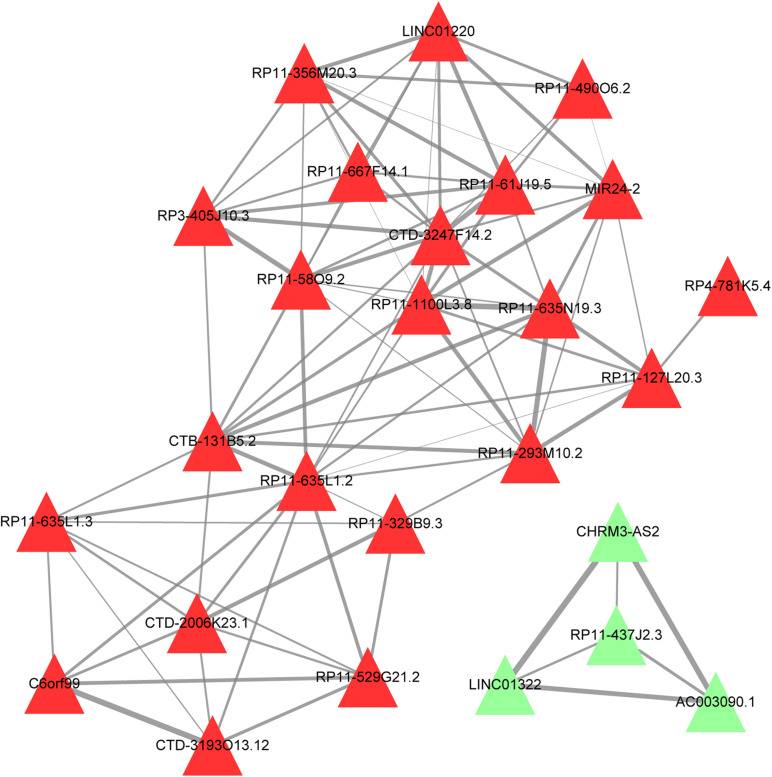
Function similarity network of lncRNAs. Red triangle, upregulated lncRNA; green triangle, downregulated lncRNAs; the thicker the connection line, the more similar the functions between the lncRNAs.

For the results obtained from GOSemSim, a Wang score >0.8 and Resnik score >0.5 were considered to have functional similarity between lncRNAs. The constructed functional similarity network showed that the upregulated CTB-131B5.2, RP11-635L1.2, and RP11-329B9.3 were linked with other lncRNAs in function (Figure 4).

### miRNA Prediction and ceRNA Construction

**FIGURE 5:**
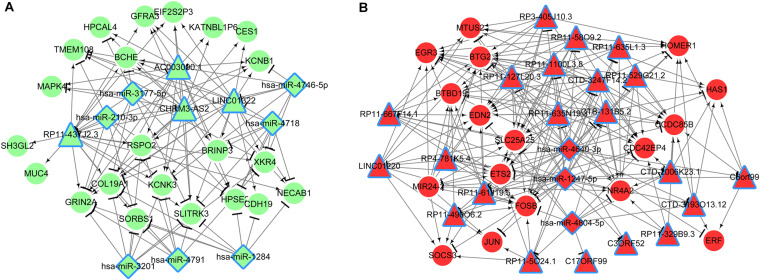
ceRNA networks (**A**, C2; **B**, C3) based on miRNA-lncRNA, lncRNA-mRNA, and miRNA-mRNA interactions. Triangle, lncRNA; rhomboid, miRNA; circle, mRNA; red, upregulated; green, downregulated.

Based on Figure 3A, the miRNAs were predicted for mRNAs in lncRNA-mRNA network. Results showed that seven miRNAs were predicted for mRNAs of C2 and three miRNAs were predicted for those of C3. No miRNAs were predicted for mRNAs in C1 network. Based on the miRanda database, nine miRNA-lncRNA interactions were predicted in C2 and 36 were predicted in C3. Finally, combining the miRNA-lncRNA, miRNA-mRNA and lncRNA-mRNA interactions, two ceRNA networks were constructed (Figure 5). The network of C2 consisted of 4 lncRNAs, 7 miRNAs, 22 mRNAs, and 115 interaction pairs. The C3 network included 21 lncRNAs, 3 miRNAs, 17 mRNAs, and 210 interaction pairs.

### DE Protein Selection and Bioinformatics Analysis

For proteomics analysis, a total of 4546 proteins were obtained by searching in the UniProt database. After differential expression analysis, 256 DE proteins were identified (Supplementary Table 3).

**FIGURE 6:**
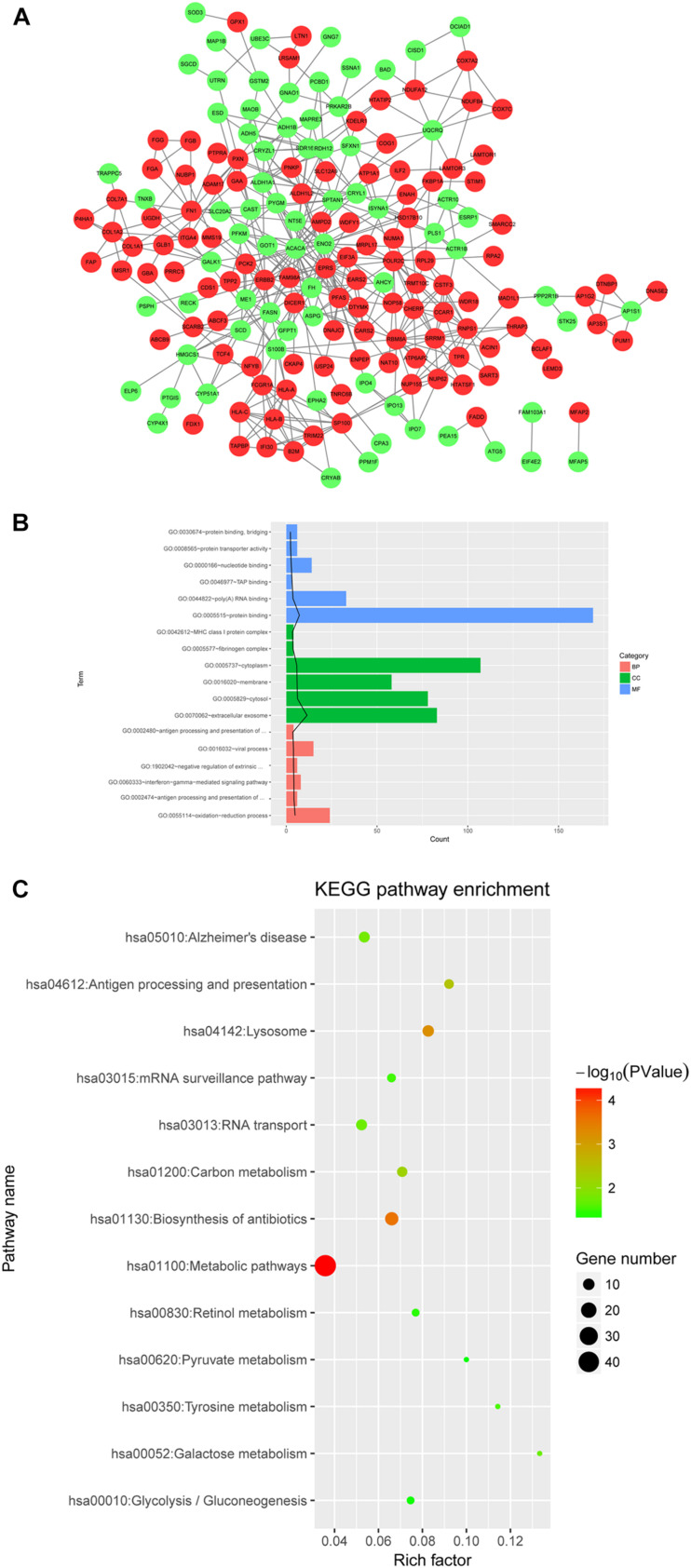
**(A)** PPI network of differentially expression proteins; **(B,C)** GO and KEGG pathway analyses for differentially expressed proteins. The top 6 GO terms ranked according to the increasing *p* values are listed. The black line stands for −log_10_ (*P* value) for each term. Red, upregulated protein; green, downregulated protein.

PPI network was constructed with protein pairs (Figure 6A). In this network, acetyl-CoA carboxylase alpha (*ACACA*), enolase 2 (*ENO2*), phosphoribosylformylglycinamidine synthase (*PFAS*), glutamyl-prolyl-tRNA synthetase (*EPRS*), spectrin alpha, non-erythrocytic 1 (*SPTAN1*), RNA polymerase II subunit C (*POLR2C*), RNA binding motif protein 8A (*RBM8A*), Erb-B2 receptor tyrosine kinase 2 (*ERBB2*), S100 calcium binding protein B (*S100B*), and dicer 1, ribonuclease III (*DICER1*) were in the top 15 genes for three topological properties.

GO analysis revealed that the DE proteins were closely related to oxidation-reduction process, protein binding, and extracellular exosome (Figure 6B). Moreover, the DE proteins were significantly involved in hsa01100:metabolic pathways, and hsa01130:biosynthesis of antibiotics (Figure 6C).

### Conjoint Analysis of Proteome and Transcriptome Data

**FIGURE 7:**
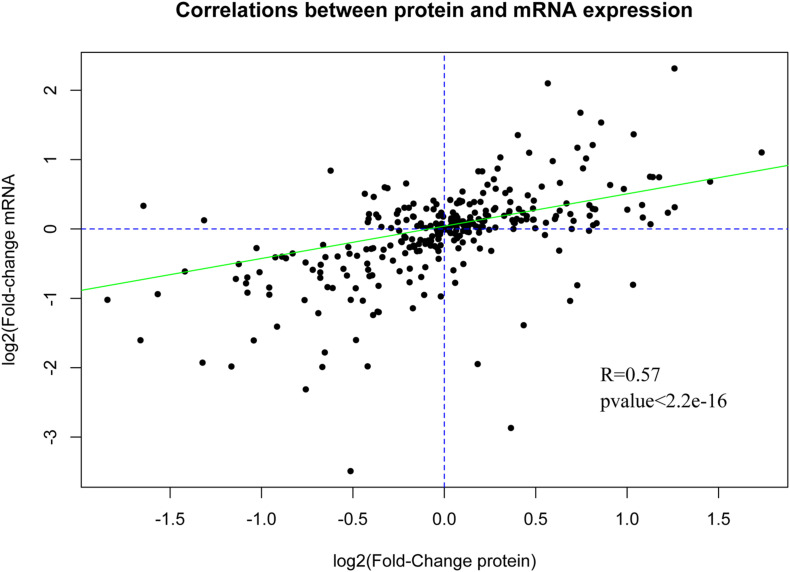
Correlation between the mRNA and protein expression levels.

The DE genes and DE proteins with *p* < 0.05 identified above were subjected to Pearson correlation analysis. The expression correlation analysis showed that the *R* values of most genes and proteins ranged from 0.7 to 1.0, suggesting that most genes and proteins had positive correlation in expression level. In addition, analysis of the correlation of the fold-change between DE mRNAs and DE proteins also showed positive correlation with *R* = 0.57 and *p* value <2.2e-16 (Figure 7), suggesting the consistency of gene and protein expression. Furthermore, Venn analysis identified 11 overlapped proteins/mRNAs [MAOB, Fc fragment of IgG binding protein (FCGBP), OCIA domain containing 2 (OCIAD2), collagen type I alpha 1 chain (COL1A1), COL7A1, glutamine-fructose-6-phosphate transaminase 1 (GFPT1), asparaginase (ASPG), proliferation and apoptosis adaptor protein 15 (PEA15), SDR16C5, secernin 1 (SCRN1), and microfibril associated protein 2 (MFAP2)]. Correlation analysis showed that there were positive correlations between protein and mRNA for FCGBP, MAOB and PEA15 (Table 3).

**TABLE 3:** Correlation analysis results of overlapped genes/proteins.

Uniprot_id	Gene_names	Rho	Description
A0A087WXI2	FCGBP	0.838689066	IgGFc-binding protein OS = Homo sapiens GN = FCGBP PE = 1 SV = 1
P27338	MAOB	0.78397768	Amine oxidase [flavin-containing] B OS = Homo sapiens GN = MAOB PE = 1 SV = 3
Q15121	PEA15	0.828973806	Astrocytic phosphoprotein PEA-15 OS = Homo sapiens GN = PEA15 PE = 1 SV = 2

### RT-PCR Validation

**FIGURE 8:**
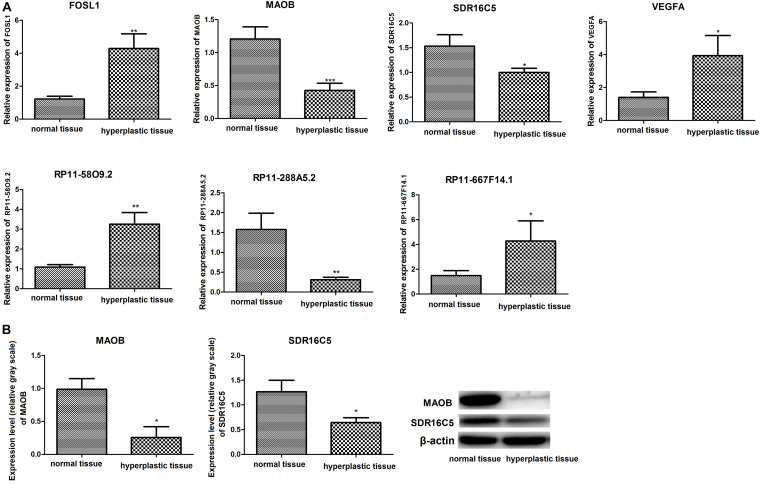
**(A)** Relative expression of *VEGFA*, *FOSL1*, *MAOB*, *SDR16C*, RP11-58O9.2, RP11-667F14.1, and RP11-288A5.2 detected by RT-PCR analysis; **(B)** Protein levels of MAOB and SDR16C detected by western blot. *p < 0.05, compared to normal tissues; **p < 0.01, compared to normal tissues.

The expression levels of RP11-58O9.2, RP11-667F14.1, *VEGFA*, and *FOSL1* in hyperplastic tissues were significantly higher than in normal tissues, which was consistent with the differential protein analysis above (*P* < 0.05). Additionally, compared to normal tissues, RP11-288A5.2, *MAOB*, and *SDR16C5* were significantly downregulated in hyperplastic tissues, which were in accordance with the prediction analysis (*P* < 0.05) (Figure 8A). However, CTD-2350J17.1 was not detected in hyperplastic tissues.

### Western Blotting Validation

The protein expression levels of MAOB and SDR16C5 are shown in Figure 8B. Compared with normal tissues, the two proteins above were downregulated in hyperplastic tissues.

## Discussion

Currently, esophageal stents have been increasingly used for palliation of malignant dysphagia and are the most common means of palliation (Sharma et al., 2002; Sharma and Kozarek, 2010). Nevertheless, tissue benign hyperplasia-induced stent restenosis is a common complication influencing the long-term effect of stent placement, however, no methods have been developed to overcome this problem. In this study, we combined high-throughput sequencing and proteomics technologies to explore the genetic and protein markers associated with benign hyperplasia caused by restenosis after esophageal stent placement.

A previous study reported that restenosis after percutaneous intervention is characterized by growth factor release, platelet aggregation, inflammatory cell infiltration, extracellular matrix remodeling medial, and smooth muscle and endothelial cell proliferation and migration (Marx et al., 2011). VEGF is a growth factor that induces the proliferation and migration of vascular endothelial cells (Leung et al., 1990). Additionally, Luttun et al. (2002) reported that VEGF may promote the development of in-stent restenosis via proinflammatory effects. A recent study showed that VEGF was upregulated in a group of patients who developed in-stent restenosis (Katsaros et al., 2013). In our study, VEGFA, a member of the VEGF growth factor family, was upregulated in hyperplasia tissues compared to normal tissues. Shu et al. (2014) suggested that VEGFA stimulates endothelial cell proliferation. Furthermore, higher VEGFA levels after percutaneous coronary intervention was reported to be associated with restenosis (Katsaros et al., 2013). Therefore, VEGFA may be a key marker of benign hyperplasia-induced restenosis.

In this study, *MAOB*, *SDR16C5* and *FOSL1* were found to be involved in oxidation-reduction associated functions, such as GO:0055114∼oxidation-reduction process (*MAOB*, and *SDR16C5*), GO:0016491∼oxidoreductase activity (*MAOB*), and GO:0042542∼response to hydrogen peroxide (*FOSL1*). Specifically, MAOB and SDR16C5 were predicted in both the transcriptome and proteomics data and presented positive correlations in mRNA and protein expression. It has been suggested that restenosis is a variable combination of *de novo* proliferative and remodeling processes with neoplastic features, as well as homeostatic repair of the vessel wall (Libby and Tanaka, 1997). Therefore, vessel injury repair may be a key determinant of restenosis. Stone and Collins (2002) have reported that a low concentration of hydrogen peroxide stimulates the proliferation and migration of endothelial cells. Additionally, oxidation-reduction process plays a prominent role in tissue injury and vascular cell signaling, which is involved repairing vessel injury (Azevedo et al., 2000). Therefore, *MAOB*, *SDR16C5*, and *FOSL1* may play key roles in esophageal restenosis involving oxidation-reduction processes.

In the constructed lncRNA-mRNA network, RP11-58O9.2 regulated the largest number of target genes. Additionally, RP11-667F14.1 regulated more than 20 targets. Interestingly, *FOSL1* was a common target of the two lncRNAs, suggesting that RP11-58O9.2 and RP11-667F14.1 play significant roles in hyperplastic tissue development by regulating *FOSL1*. Functional analysis showed that RP11-58O9.2 is involved in the IL-17 signaling pathway. Interleukin (IL)-17 may stimulate several types of cells to secrete multiple proinflammatory mediators. Local production of IL-17 may cause site-specific influx and activation of inflammatory cells (Fossiez et al., 1998). Importantly, studies have supported a key role for inflammatory cells in the restenosis process (Welt and Rogers, 2002). Additionally, RP11-58O9.2 and RP11-667F14.1 were significantly enriched in the pathway of HTLV-I infection. A previous study has investigated the correlation between HTLV-1 and esophageal squamous cell carcinoma (Mirsadraee et al., 2007), but no correlation is found. In this study, we did not test HTLV-1 in patients. Thus, the role of HTLV-1 infection in esophageal cancer needed to be further investigated. Taken together, we speculated that stent restenosis induced by hyperplasia may be modulated via the regulatory role of RP11-58O9.2 and RP11-667F14.1 in *FOSL1* expression as well as the IL-17 signaling pathway.

Furthermore, both RP11-288A5.2 and CTD-2350J17.1 regulated *MAOB* with the top two lower adjusted *p* values. Specifically, the expression of RP11-288A5.2 was verified. As described above, *MAOB* was enriched in functions associated with oxidation-reduction process. Additionally, this protein was involved in the GO:0032496∼response to lipopolysaccharide (LPS). LPS is a large molecule consisting of a lipid and polysaccharide, which acts as an endotoxin to elicit strong immune responses in animals. Moreover, LPS induces extracellular matrix degradation and stimulates the production of various cytokines (Aota et al., 2006). Forrester et al. (1991) suggested that there were three phases in the restenosis process, including an inflammatory phase, cellular proliferation phase, and remodeling of extracellular matrix protein synthesis phase, which indicates an important role for extracellular matrix remodeling in restenosis. Additionally, the extracellular matrix is the basis for lesion growth. The aortic hyperplasia is related to extracellular matrix deposition (Dao et al., 2001). Therefore, RP11-288A5.2 and CTD-2350J17.1 may be associated with restenosis by participating in extracellular matrix remodeling. The expression and role of CTD-2350J17.1 in benign hyperplasia-induced restenosis requires further analysis.

Besides, the pathway analysis showed that DE proteins in hyperplasia tissues compared with normal tissues in patients with esophageal cancer were significantly enriched in the biosynthesis of antibiotics pathway. A previous report suggested that the biosynthesis of antibiotics and other secondary metabolites was controlled by inorganic phosphate (Martín, 2004). The alterations in carbohydrate metabolism are present in endometrial hyperplasia and endometrial carcinoma patients (Benjamin and Casper, 1966). The level of inorganic phosphate in blood plasma is related with glucose metabolism underlying glucose use. The level of phosphate has been considered as an index of peripheral use of glucose. In addition, the global gene analysis indicated the antibiotic biosynthetic pathways in *Streptomyces* (Huang et al., 2001). In the present study, after sequencing, the data of each sample were subjected to comparative analysis and the results showed that all the data were reliable, which suggested that there was no bacterial contamination in the process of experiments. Thus, we suggested that the dysregulation of biosynthesis of antibiotics pathway may be related with the glucose metabolism in the hyperplasia tissues.

In conclusion, we combined transcriptome and proteomics data to investigate the critical genes/proteins associated with benign hyperplasia-induced restenosis in patients with esophageal cancer. DE *VEGFA*, *FOSL1*, *MAOB*, *SDR16C5*, RP11-58O9.2, RP11-667F14.1, and RP11-288A5.2 in hyperplastic tissues may serve as genetic targets for preventing stent restenosis in esophageal cancer. However, the sample size used in this study was a little small, so we will collect more samples to confirm our results in the future study.

## Materials and Methods

### Patients and Samples

From April to July in 2017, five patients (Supplementary Table 1) with esophageal cancer admitted to our hospital were enrolled in the study. These patients were subjected to ^125^I particle esophagus stent placement for palliation of dysphagia due to esophagus cancer and presented with tissue hyperplasia around the perforation of the stent several months after stent placement. Five pairs of hyperplastic tissues and adjacent non-hyperplastic tissues (normal tissues) were collected during surgery, among which four pairs were used for lncRNA sequencing and all five pairs were used for proteomics. Our study was approved by the ethics committee of our hospital. All patients provided informed consent before the experiments.

### Total RNA Extraction and RNA Sequencing

Total RNA was extracted from four pairs of hyperplastic tissues and normal tissues using TRIzol reagent (Takara, Shiga, Japan) according to the manufacturer’s instructions. The quality and concentration of RNA samples were, respectively, determined with a NanoDrop spectrophotometer (Wilmington, DE, United States) and Qubit 2.0 Fluorometer (Life Technologies, Carlsbad, CA, United States). The quality of RNA samples is shown in Supplementary Table 2. RNA integrity was detected with an Agilent Bioanalyzer 2100 (Agilent Technologies, Santa Clara, CA, United States). Ribosomal RNA was removed from total RNA samples by using a Ribo-zero rRNA Removal Kit (EPICENTRE, Madison, WI, United States). Strand-specific libraries were constructed by the dUTP method using the NEB Next Ultra Directional RNA Library Prep Kit for Illumina (NEB, Ipswich, MA, United States). The libraries were sequenced on an Illumina HiSeq 2500 platform with 150-base pair single-end reads.

### Quality Control and Reference Genome Alignment

Trimmomatic (version 3.6) (Bolger et al., 2014) tool was used for quality control of raw reads. Unreliable bases and low-quality reads were removed to obtain clean reads. The clean reads were mapped to the human genome (GRCh38.p7, GENCODE) (Harrow et al., 2012) using TopHat2 (version 2.1.0) (Trapnell et al., 2009) and the lncRNA and mRNA information was obtained.

The sequencing data are deposited in the NCBI Sequence Read Archive (SRA) database with the accession number PRJNA544132.

### Analysis of Gene Expression Levels and Differentially Expressed (DE) Genes

The gene expression level was determined by estimating the reads located in genomic regions or gene exon regions. Based on the human genome annotation information in the GENCODE database, the count of reads mapped to the genes was obtained using HTSeq (version 0.6.1p2) (Anders et al., 2015), which was then normalized using the counts per million method. Additionally, genes with a CMP value of <0.1 in at least three samples were defined as low expression abundance genes. The obtained genes were divided into lncRNA and mRNA according to the gene type in the annotation information.

Differential expression analysis for lncRNAs and mRNAs was performed using likelihood ratio tests in edgeR of R (Lun et al., 2016). DE lncRNAs and mRNAs between paired hyperplastic tissues and normal tissues were analyzed. Moreover, RNAs with low expression abundance were deleted before DE analysis. A |logFC (fold change)| > 1 and *p* value <0.05 were used as thresholds of DE analysis.

### Functional Enrichment Analysis of DE mRNAs

The DE mRNAs were subjected to Gene Ontology (GO) function and Kyoto Encyclopedia of Genes and Genomes (KEGG) pathway analyses by using the DAVID (version 6.8) online tool (Huang et al., 2008). The significantly enriched GO terms and pathways were selected with the thresholds of a count ≥2 and *p* value <0.05.

### Protein-Protein Interaction (PPI) Network Analysis

The PPIs of DE mRNAs were analyzed using the online tool STRING (Szklarczyk et al., 2015). Required confidence (combined score) > 0.4 was selected as the threshold of PPI. Next, the network based on the PPI was constructed using Cytoscape (Shannon et al., 2003), and topological properties [degree centrality (Opsahl et al., 2010), betweenness centrality (Cukierski and Foran, 2008), and closeness centrality (Du et al., 2015)] of the network were analyzed using CytoNCA (Tang et al., 2015) in Cytoscape to identify hub nodes in the PPI network.

### LncRNA-mRNA Interaction Network Analysis

The correlations between the obtained DE lncRNAs and mRNAs were analyzed by calculating the Pearson correlation coefficients (Pearson, 2006). The *p* values were adjusted using the Benjamini and Hochberg (1995) method. An adjusted *p* value <0.01 indicated a correlation between DE lncRNAs and mRNAs and the mRNAs were considered as potential target genes of lncRNAs. Finally, the lncRNA-target gene regulatory network was constructed using Cytoscape software (Shannon et al., 2003).

### Function Analysis of lncRNAs

The functions of DE lncRNAs were predicted through GO and KEGG analyses of their target genes. In this study, lncRNAs with ≥15 targets were subjected to cellular component (CC), molecular function (MF), biological process (BP), and pathway prediction using clusterProfiler (Yu et al., 2012) with an adjusted *p* value <0.05.

The GO function (BP) similarity between lncRNA was determined using Resnik method [Resnik method (Poesio, 1999) and Wang method (Wang et al., 2007)] provided by GOSemSim (Yu et al., 2010). Resnik method is an information content-based method depending on the frequencies of the two GO terms involved and that of their closest common ancestor term in a specific corpus of GO annotations; the Wang method is a graph-based method that uses the topology of the GO graph structure to compute semantic similarity.

### miRNAs Prediction and Competitive Endogenous RNA (ceRNA) Network Construction

Based on the lncRNA-mRNA interactions, we predicted the miRNAs that regulated the mRNAs using TargetScan_microRNA_2017 in Enrichr (Chen et al., 2013). The miRNA-mRNA regulatory pair with a *p* value <0.01 was selected. Next, the interactions between the predicted miRNAs and obtained lncRNAs were predicted using miRanda (Enright et al., 2003). If there was a binding site between an miRNA and obtained lncRNA, we considered that there was a regulatory interaction between these factors. Based on the miRNA-lncRNA, miRNA-mRNA, and lncRNA-mRNA interactions, a ceRNA network was constructed using Cytoscape software.

### TMT Quantitative Proteomics

Proteins between five pairs of hyperplastic tissues and normal tissues were analyzed by tandem mass tag technology. Briefly, protein was extracted from five pairs of tissues using RIPA lysis buffer (Beyotime Biotechnology, Shanghai, China) and quantified using the BCA^TM^ Protein Assay Kit (Pierce, Madison, WI, United States). The quality of protein samples was evaluated by 12% sodium dodecyl sulfate-polyacrylamide gel electrophoresis. The qualified proteins were subjected to reductive alkylation. Then trypsin was added at an enzyme to protein ratio of 1:50 (w/w) and incubated at 37°C overnight. Following that the peptides were dissolved in 0.5 M tetraethyl-ammonium bromide and then labeled with TMT. The five samples in each group were pooled and primarily separated with the RP-C18 column, followed by liquid chromatography coupled with tandem mass spectrometry (LC-MS/MS) analysis. Liquid chromatography separation was carried out using a reversed-phase column (75 μm × 25 cm; Thermo, United States). The columns were connected to an EASY-n LC 1200 system (Thermo, United States). Total 5 μl of sample was loaded onto the column with starting mobile phase of 2% ACN and 0.1% formic acid, followed by 80% ACN with 0.1% formic acid for 120 min. The flow rate was held at 300 nL/min. Mass spectra were acquired on a Q-Exactive (Thermo, United States) mass spectrometer in a data-dependent manner. Peptide fragmentation was performed via higher-energy collision dissociation with a resolution of 70,000 followed by 35,000, and the exclusion duration was 18 s. The scan range ranged from 350 to 1300 m/z.

### Protein Identification and Quantification

LC-MS/MS data were analyzed using Proteome Discoverer^TM^ Software 2.1 interfaced with UniProt uniprot-proteome-UP000005640-Homosapiens database (The UniProt Consortium, 2017)^1^ at 20170912. In order to avoid precursor ion interference, the trypsin was added at an enzyme to protein ratio of 1:50 (w/w) and incubated at 37°C overnight. The polypeptide samples were re-dissolved in UPLC loading buffer and separated by high-performance liquid chromatography with C18 reverse phase column. Total 71591 human protein sequences in FASTA format were searched. The searched parameters included two maximal missed cleavages, fixed modifications [Carbamidomethyl (C), TMT 6plex(K), TMT 6plex (N-Terminus)], variable modifications [Oxidation(M), Acetyl (Protein N-Terminus)], Mass tolerance for precursor ions (20 ppm), Mass tolerance for fragment ions (0.02 Da). Percolator node within Proteome Discoverer were used to calculate the false discovery rates (FDRs) for Peptide-spectrum matches (PSMs). PSMs were filtered by 7 minimal peptide length, mass accuracy (∼2.5 ppm) and matching scores to achieve 1% protein FDR.

All the peptide sequences and proteins identification were listed in the Supplementary Files 1, 2. The mass spectrometry proteomics data have been deposited to the ProteomeXchange Consortium via the PRIDE (Perez-Riverol et al., 2018) partner repository with the dataset identifier PXD014242.

### DE Protein Identification and Bioinformatics Analysis

The quantitative data of protein abundance in the two groups of matched samples were normalized with loess method (Cleveland et al., 1992; Linton, 2018) and then analyzed by paired sample *t-*test to screen for DE proteins. Proteins with *p* value <0.05 were considered differentially expressed.

DE proteins were then subjected to PPI analysis using STRING, as well as GO and KEGG pathway analyses with DAVID 6.8 as described above.

### Conjoint Analysis of Proteome and Transcriptome Data

Based on the obtained proteome and transcriptome data, we selected the common proteins/genes and calculated the correlation between the proteins and genes based on expression levels using the Pearson correlation coefficient. Results with *p* < 0.05 were considered reliable. Additionally, we performed VENN analysis for the DE proteins and mRNAs to screen for overlapped DE proteins/mRNAs. The Pearson correlation between the overlapped DE proteins and mRNAs in expression abundance was also analyzed.

### RT-PCR Validation

Total RNA was isolated from hyperplastic tissues and normal tissues (50–100 mg) using the TRIzol method. After quality and purity evaluation, the reverse transcription reaction was performed with 0.5 μg RNA by using PrimeScript RT Master Mix (RR036A, Takara). Real-time PCR analysis was performed in a reaction volume of 20 μL (10 μL SYBR Premix EX Taq (2×), 1 μL forward primer, 1 μL reverse primer, and 8 μL cDNA). PCR amplification was performed as follows: 50°C for 3 min, 95°C for 3 min, and 40 cycles of 95°C for 10 s and 60°C for 30 s. The expression levels of RP11-288A5.2, RP11-58O9.2, RP11-667F14.1, CTD-2350J17.1, monoamine oxidase B (*MAOB*), short chain dehydrogenase/reductase family 16C member 5 (*SDR16C5*), vascular endothelial growth factor A (*VEGFA*), and FOS like 1, AP-1 transcription factor subunit (*FOSL1*) were detected. Glyceraldehyde 3-phosphate dehydrogenase (GAPDH) was used as an internal control. The primer sequences are listed in Table 4.

**TABLE 4:** Primer sequence for RT-PCR.

Primer	Sequences (5′-3′)
GAPDH-hF	TGACAACTTTGGTATCGTGGAAGG
GAPDH-hR	AGGCAGGGATGATGTTCTGGAGAG
RP11-288A5.2-hF	CCCACTTTCCTCCCATAA
RP11-288A5.2-hR	CCTAATCAGCAGCCGTTTC
CTD-2350J17.1-hF	CCCTTATTTCCTGCTTC
CTD-2350J17.1-hR	CAGAGTTGAATGGTGCTT
RP11-58O9.2-hF	TGGGCTGAGCTATGTGAC
RP11-58O9.2-hR	AGTTCTCCGCTAAACCTTC
RP11-667F14.1-hF	AGGAAGTGTCACGGTCTCG
RP11-667F14.1-hR	ATCCCAGTGGTTGTAGGTGTT
VEGFA-hF	GCCTTGCCTTGCTGCTCTAC
VEGFA-hR	CTCGATTGGATGGCAGTAGC
FOSL1-hF	CAGGCGGAGACTGACAAACTG
FOSL1-hR	TCCTTCCGGGATTTTGCAGAT
MAOB-hF	GGCGGCATCTCAGGTATGG
MAOB-hR	GGTCTCCAATCCTAGCTCCTTG
SDR16C5-hF	TATACCTGCGATTGCAGCCAA
SDR16C5-hR	CGATTCCGGCATTGTTGATTAGG

### Western Blotting Validation

Proteins were extracted from hyperplastic tissues and normal tissues with RIPA buffer (P0013B; Beyotime, Shanghai, China). Protein concentrations were measured with BCA method using a BCA protein assay kit (PL212989; Thermo, United States). After denaturation, protein samples were subjected to SDS-PAGE and blotted onto polyvinylidene fluoride membranes (IPVH00010; Millipore, United States). Non-specific binding sites were blocked with 5% skim milk. Following washing with 1 × PBS-T (1000 mL 1 × PBS + 1 mL Tween-20) for three times, the membranes were incubated with primary antibodies [1:1000; MAOB (ab88510; Abcam, United States) and SDR16C5 (Cat #PA5-31421; Thermo)], or β-actin (Sc-47778; Santa Cruz Biotechnology, United States) overnight at 4°C, followed by washing six times in 1 × PBS-T. Then the membranes were incubated with horseradish peroxidase conjugated secondary antibodies [anti-mouse (1:5000) or anti-rabbit IgG (1:10000)] for 2 h at 37°C and washed for six times with 1 × PBS-T. Finally, the membranes were visualized by ECL detection (Millipore, United States).

### Statistical Analysis

The experiments of RT-PCR and western blotting were repeated three times. Data were shown as mean ± standard deviation (SD). GraphPad Prism 5 (San Diego, CA) was used to analyze the data from this study. Student’s *t*-test was used for comparison. Statistical significance was considered for *p*-value >0.05.

## Data Availability Statement

The sequencing data have been deposited in the NCBI Sequence Read Archive (SRA) database with the accession number PRJNA544132. The mass spectrometry proteomics data have been deposited to the ProteomeXchange Consortium via the PRIDE partner repository with the dataset identifier PXD014242.

## Ethics Statement

The studies involving human participants were reviewed and approved by Ethics Committee of Tongren Hospital, Shanghai Jiao Tong University School of Medicine. The patients/participants provided their written informed consent to participate in this study. Written informed consent was obtained from the individual(s) for the publication of any potentially identifiable images or data included in this article.

## Author Contributions

AM and XZ: conception and design of the research, obtaining funding, and revision of manuscript for important intellectual content. MS, SW, and XY: acquisition of data. MS, BL, and XY: analysis and interpretation of data. MS and XY: statistical analysis. LW and SS: drafting the manuscript. All authors read and approved the final manuscript.

## Conflict of Interest

The authors declare that the research was conducted in the absence of any commercial or financial relationships that could be construed as a potential conflict of interest.

## Footnotes

1 http://www.uniprot.org/proteomes/UP000005640

## Supplementary Material

Supplementary Table 1 Patient information.

Supplementary Table 2 The quality of RNA samples.

Supplementary Table 3 DE mRNAs, lncRNAs and proteins.

Supplementary File 1 Proteins identification.

Supplementary File 2 The peptide sequences.
