# Supplementary material for: Identification of Critical Genes and Proteins for Stent Restenosis Induced by Esophageal Benign Hyperplasia in Esophageal Cancer
Source: Front Genet. 2020 Dec 17;11:563954. doi: 10.3389/fgene.2020.563954 (PMC7773907; doi:10.3389/fgene.2020.563954)
Supplement: Supplementary Table 1 — Patient information. [file Table_1.DOCX]

Table S1 Patient information.

| **ID** | **case** |
| --- | --- |
| 7X/7Y | Male; 44 years old; esophageal cancer (clinic stage II) |
| 8X/8Y | Female; 71 years old; esophagus cancer (clinic stage III) |
| 10X/10Y | Female; 69 years old; esophagus cancer (clinic stage I) |
| 11X/11Y | Female; 65 years old; esophagus cancer (clinic stage III) |
| 12X/12Y | Female; 57 years old; esophageal cancer (clinic stage II) |
