# Supplementary material for: Identification of Critical Genes and Proteins for Stent Restenosis Induced by Esophageal Benign Hyperplasia in Esophageal Cancer
Source: Front Genet. 2020 Dec 17;11:563954. doi: 10.3389/fgene.2020.563954 (PMC7773907; doi:10.3389/fgene.2020.563954)
Supplement: Supplementary Table 2 — The quality of RNA samples. [file Table_2.DOC]

**Table S2** The quality of RNA samples.

| Sample | Extraction ID | Concentration (ng/μL) | Volume (μL) | Total (μg) | OD260/280 | OD260/230 | Type | 28s/18s | RIN | Result |  |
| --- | --- | --- | --- | --- | --- | --- | --- | --- | --- | --- | --- |
| 7Y-hyperplastic tissue | TZTR170801250 | 246 | 55 | 13.53 | 1.952 | 1.482 | lncRNA | 1.1 | 7.7 | A |  |
| 8Y-hyperplastic tissue | TZTR170805048 | 286 | 32 | 9.152 | 1.910 | 1.568 | lncRNA | 1 | 7.2 | A |  |
| 10Y-hyperplastic tissue | TZTR170801252 | 168 | 55 | 9.24 | 1.936 | 1.750 | lncRNA | 1 | 6.9 | A |  |
| 11Y-hyperplastic tissue | TZTR170801253 | 155 | 55 | 8.525 | 2.000 | 1.702 | lncRNA | 1 | 7.3 | A |  |
| 7X-normal tissue | TZTR170801255 | 238 | 55 | 13.09 | 2.052 | 0.810 | lncRNA | 1.4 | 8.4 | A |  |
| 8X-normal tissue | TZTR170801256 | 156 | 55 | 8.58 | 1.950 | 1.50 | lncRNA | 1.1 | 7.3 | A |  |
| 10X-normal tissue | TZTR170801257 | 300 | 55 | 16.5 | 1.948 | 1.852 | lncRNA | 1.1 | 7.4 | A |  |
| 11X-normal tissue | TZTR170801258 | 121 | 55 | 6.655 | 1.838 | 1.700 | lncRNA | 1.2 | 7.2 | A |  |
